# Renal Resistive Index on Admission Predicts and Mediates Acute Kidney Injury: A Prospective Observational Study from a Greek Intensive Care Unit

**DOI:** 10.3390/jcm15072649

**Published:** 2026-03-31

**Authors:** Stelios Kokkoris, Ioannis Melissovas, Georgia Fotopoulou, Ioannis Poularas, Eleni Margioula, Ilias Premetis, Dimitrios Tsilivarakis, Sofia Mavromati, Stavros Spiliopoulos, Christina Routsi

**Affiliations:** 1 First Department of Critical Care Medicine, School of Medicine, National and Kapodistrian University of Athens, Evangelismos Hospital, 106 76 Athens, Greece; skokkoris2003@yahoo.gr (S.K.); grg_fotopoulou@yahoo.com (G.F.); poularas@otenet.gr (I.P.); margioula@hotmail.com (E.M.); ilias.7prem@gmail.com (I.P.); tsilivarakisd@gmail.com (D.T.); sofmavro@gmail.com (S.M.); 2 Department of Radiology, Evangelismos Hospital, 106 76 Athens, Greece; jmelissovas@gmail.com; 3 Second Department of Radiology, Interventional Radiology Unit, School of Medicine, National and Kapodistrian University of Athens, “Attikon” University General Hospital, 124 62 Athens, Greece; stavspiliop@med.uoa.gr

**Keywords:** renal resistive index, acute kidney injury, critical illness, Doppler ultrasonography, intensive care unit, observational study

## Abstract

**Background/Objectives**: The renal resistive index (RRI) has emerged as an early marker of renal vascular resistance. The purpose of this study was to investigate the association between RRI on intensive care unit (ICU) admission and the development of acute kidney injury (AKI) in a general ICU population, and to assess its predictive accuracy. **Methods**: This prospective observational study was conducted in a multidisciplinary ICU. Consecutive mechanically ventilated adults were enrolled; RRI was measured within 24 h of admission after hemodynamic stabilization. AKI was defined by Kidney Disease: Improving Global Outcomes (KDIGO) criteria within seven days. Multivariable regression, receiver operating characteristic (ROC), reclassification, and mediation analyses were performed. **Results**: A total of 181 patients were included. AKI occurred in 36%. Median RRI was 0.73 (0.65–0.80). RRI correlated with age, acute physiology and chronic health evaluation (APACHE) II and sequential organ failure assessment (SOFA) scores, lactate, and glomerular filtration rate (GFR) (all *p* < 0.001). In multivariable analysis, RRI was the only independent predictor of AKI (OR 2.86 per 0.05 increase, 95% CI 1.64–4.98, *p* = 0.001). It was also associated with an increased likelihood of presenting with a more severe AKI stage. RRI showed high discriminative ability (AUC = 0.89, 95% CI 0.84–0.94); the optimal cut-off was 0.77 (sensitivity 0.83, specificity 0.82). Adding RRI to a clinical model improved prediction (ΔAUC *p* = 0.049; net reclassification index (NRI) = 0.52, *p* < 0.001). Mediation analyses showed that RRI significantly mediated the effects of hypertension and low baseline GFR on AKI risk. Subgroup analyses confirmed consistent predictive performance across age, lactate, and sepsis categories. **Conclusions**: RRI is an independent early predictor of AKI and its severity, as well as a mediator of both hypertension and low GFR, regarding their effect on AKI development in ICU patients. RRI could serve as an early bedside marker of renal perfusion impairment in critically ill patients, guiding strategies aimed at reducing the risk of AKI.

## 1. Introduction

The global incidence of acute kidney injury (AKI) in the general population is estimated at 114–174 cases per 10,000 person-years, with approximately 13.3 million cases worldwide in 2017 [1]. AKI is a multifactorial condition affecting 10–15% of hospitalized patients and more than 50% of patients in the intensive care unit (ICU), and is associated with increased morbidity and mortality [1,2]. There is growing evidence that the burden of AKI extends beyond the acute phase, with progression to chronic kidney disease (CKD), enhanced risk of cardiovascular complications, and long-term mortality [3]. Despite numerous studies, the early identification of patients at risk for AKI remains a major clinical challenge.

The renal resistive index (RRI), a non-invasive Doppler ultrasound parameter derived from intrarenal arterial blood flow velocities, has emerged as a potential early marker of renal vascular resistance and perfusion abnormalities. This method is simple, rapid, noninvasive, and repeatable, as it determines RRI by measuring systolic and diastolic blood flow velocities from Doppler waveforms obtained in the intrarenal arcuate or interlobar arteries, and has been increasingly recognized as a valuable tool for assessing renal perfusion in critically ill patients [4].

Currently, RRI’s growing body of research in critically ill populations has identified RRI as a promising marker for detecting patients at risk of AKI onset [5,6], AKI persistence [7,8], and for predicting clinical outcomes, such as mortality [9]. However, its prognostic value in a general intensive care unit (ICU) population that includes patients with diverse etiologies of critical illness remains less well characterized.

Conventional markers, such as serum creatinine and urine output, often rise only after significant kidney injury has occurred, underscoring the need for earlier, more sensitive indicators of renal dysfunction. This delay in detection creates a ‘therapeutic window’ where interventions are often too late. RRI potentially fills this gap by identifying hemodynamic changes before structural damage occurs.

Given these uncertainties, a more comprehensive evaluation of RRI’s role as a predictor of AKI in heterogeneous ICU populations is warranted. Understanding its relationship with both renal and systemic factors, as well as its potential mediating role in the pathway linking comorbidities like hypertension or impaired glomerular filtration rate (GFR) to AKI development, could improve our understanding of kidney vulnerability in critical illness. We assume that RRI could serve as an early bedside marker of renal perfusion impairment in critically ill patients, guiding strategies aimed at reducing the risk of AKI.

### Study Objectives

The overarching goal of this study is to determine whether baseline RRI can serve as a reliable early-warning bedside tool in a heterogeneous ICU population. Specifically, we aimed to investigate the following:

Primary objective: Evaluate the association between baseline RRI and the development of AKI and assess its overall predictive accuracy.

Secondary objectives: Identify correlations between RRI and systemic clinical variables; analyze the mediating role of RRI in the relationship between pre-existing comorbidities (hypertension, low GFR) and AKI; compare RRI performance across distinct patient subgroups (e.g., surgical vs. medical ICU).

## 2. Methods

### 2.1. Setting

This prospective observational study was conducted between October 2017 and September 2018, as well as between January 2023 and June 2023, in the 30-bed multidisciplinary ICU of Evangelismos Hospital, a tertiary care academic medical center. This ICU admits critically ill medical, surgical, and trauma patients. Patients with acute coronary syndromes, cardiac surgery, or organ transplantation are managed in specialized units. The study protocol was approved by the Evangelismos Hospital Ethics Committee (approval number 38/03-2017); oral informed consent was obtained from the next of kin of all participants. This study was conducted and reported in accordance with the Strengthening the Reporting of Observational Studies in Epidemiology (STROBE) guidelines for cohort studies [10].

### 2.2. Patients and Data Collection

All consecutive adult patients requiring invasive mechanical ventilation upon ICU admission were screened for eligibility. Exclusion criteria included age under 18 years; pregnancy; history of renal disease; known conditions that could alter RRI, such as renal artery stenosis, urinary obstruction or structural renal abnormalities; morbid obesity leading to poor echogenicity; increased intrabdominal pressure; readmission or transfer from another ICU; and anticipated ICU stay shorter than 48 h. Weekend admissions were not included due to the unavailability of experienced ultrasound operators. Demographic and clinical data, including admission diagnosis, comorbidities, laboratory parameters, presence of sepsis or shock, vasopressor use and dosage, development of AKI, and ICU outcome, were recorded. Disease severity was assessed on the first ICU day using the acute physiology and chronic health evaluation II (APACHE II) [11] and the sequential organ failure assessment (SOFA) scores [12]. In addition, to determine baseline renal function, admission GFR was estimated [13]. Missing values of RRI measurements were 0%, and for variables used in the analyses less than 3%.

### 2.3. Protocol and Measurements

All enrolled patients were receiving mechanical ventilation in assist control mode for acute respiratory failure from various causes. They had an arterial line and a central venous catheter in an internal jugular or subclavian vein inserted by the patients’ attending physicians as part of the routine ICU management.

Renal Doppler ultrasonography was performed within 24 h of ICU admission, after the initial hemodynamic stabilization, by experienced operators (I.M., radiologist and G.F. and I.P., intensivists) who were not involved in the patients’ management. The Vivid 7 ultrasound system (General Electric Healthcare, UK) was used. RRI was measured as previously described [14]. In brief, using a 7.5 MHz linear probe, gray-scale imaging was first performed to assess renal anatomy. Color Doppler imaging was then used to identify arcuate or interlobar arteries, followed by pulsed-wave Doppler to record the velocity–time waveform. RRI was calculated using the formula: RRI = (peak systolic velocity − end-diastolic velocity)/peak systolic velocity. For each kidney, three to five reproducible waveforms were obtained from the upper, mid, and lower poles. The final RRI was calculated as the mean of right and left kidney values. Normal values are reported between 0.60 and 0.70 [15].

Hemodynamic parameters, including heart rate and invasive systolic and diastolic blood pressure, were recorded concurrently with the RRI measurement. No fluid administration or changes in catecholamine infusion rates were allowed during measurements. Blood samples were drawn simultaneously from the arterial line and the central venous catheter around the time of ultrasonography, and were immediately analyzed (ABL 300, Radiometer; Copenhagen, Denmark).

### 2.4. Definitions

Sepsis was defined as suspected or confirmed infection accompanied by acute organ dysfunction [16]. Circulatory shock was defined as hypotension (systolic blood pressure < 90 mm Hg and/or mean arterial pressure < 65 mmHg), persisting despite adequate volume resuscitation, requiring administration of vasoactive agents [17]. AKI was defined according to the Kidney Disease: Improving Global Outcomes (KDIGO) criteria, based on serum creatinine or urine output [18]. Baseline serum creatinine was defined as the most recent value available prior to hospitalization; if unavailable, the admission value was used. The occurrence of AKI was assessed within seven days following ICU admission.

### 2.5. Statistical Analysis

We presented continuous variables as median with interquartile range (IQR) and compared them using the Mann–Whitney or Kruskal–Wallis test, as appropriate. We presented categorical variables as percentages and compared them using the chi-squared or Fisher’s exact test, as appropriate. Correlations between variables were estimated by Spearman’s rho coefficients. We estimated the association between RRI and development of AKI via logistic regression analysis. We also estimated the association between RRI and AKI stages with an ordinal multivariate logistic regression. We adjusted for various independent variables selected by least absolute shrinkage and selection operator (LASSO) regression, which is especially useful when you have a lot of predictors and want to find the most important ones while avoiding overfitting. The optimal penalty parameter (λ) was selected using 10-fold cross-validation, and the value minimizing the cross-validated binomial deviance (λ_min) was used for the final LASSO model. We performed and compared two multivariate regressions in order to delineate whether RRI remains a significant factor for AKI prediction. Firstly, we defined a reference clinical model including the same baseline variables used in the previous logistic regression. Then, a separate multivariate logistic regression analysis was conducted to assess the performance of the predictive model, after adding RRI to the reference model. The total number of AKI events used in the multivariable models was 66. Seven (reference model) and 8 (reference model + RRI model) variables were used; they were selected by the Lasso regression, and the events per variable (EPV) ratios were 9.4 and 8.3, respectively. Improvement in the model was evaluated by difference in the area under the curves (AUCs) (ΔAUC) estimated by the DeLong test. We also quantified the improvement of the new model on AKI risk prediction with the net reclassification improvement (NRI), which measures how much a new model improves the classification of individuals compared with a reference model. It assesses whether the new model more accurately reclassifies individuals into appropriate risk categories. Predicted AKI risk was categorized using thresholds at 0.1 increments (0.1, 0.2, …, 0.9), resulting in ten risk categories used to quantify patient reclassification between the reference and extended models. A positive NRI indicates that the new model improves classification compared with the reference model. We performed mediation analyses using baseline GFR and a history of hypertension as exposures, and RRI as mediator for AKI development. Mediation analysis is a statistical approach used to understand the mechanism by which an independent variable affects a dependent variable through a mediator. It decomposes the total effect into direct and indirect (mediated) effects. Mediation analysis relies on the assumption that there is no unmeasured confounding of the exposure–mediator, exposure–outcome, and mediator–outcome relationships conditional on the included covariates. To minimize potential confounding, all models were adjusted for clinically relevant variables selected a priori. However, as this was an observational study, residual confounding cannot be completely excluded. Receiver operating characteristic (ROC) curves were used to determine the cut-off value of RRI and to evaluate its predictive power for AKI occurrence. Internal validation of all model performances was performed using bootstrap resampling (1000 samples) according to Harrell’s method to estimate optimism and obtain an optimism-corrected AUC. There were small differences between the apparent and optimism-corrected AUC values, suggesting limited overfitting and stable predictive performances. All AUCs, throughout the manuscript and the tables, are presented as the corrected values. An optimal cut-off value was calculated by Youden’s index. Decision curve analysis was performed to evaluate the potential clinical utility of the RRI for predicting AKI. Net benefit was calculated across a range of clinically relevant threshold probabilities (0.01–0.50), comparing the strategy of using RRI to guide clinical decisions with the default strategies of treating all patients or treating none. Net benefit incorporates the relative consequences of false-positive and false-negative classifications and reflects the clinical value of a prediction marker across different risk thresholds. Confidence intervals were estimated using bootstrap resampling with 1000 iterations. Further prespecified stratified analyses were conducted based on age (<65 and ≥65 years), lactate level (<2 and ≥2 mmol/L), and sepsis status to assess the consistency of the prognostic value of RRI for AKI. Interactions between RRI and stratification variables were examined using likelihood ratio tests. We considered statistical significance at an α level of 0.05; all *p* values were two-sided. We conducted all statistical analyses using SPSS software version 24.0 (SPSS, Inc., Chicago, IL, USA) and R software version 4.2.1 (R Foundation for Statistical Computing).

## 3. Results

### 3.1. Study Population

**Figure 1:**
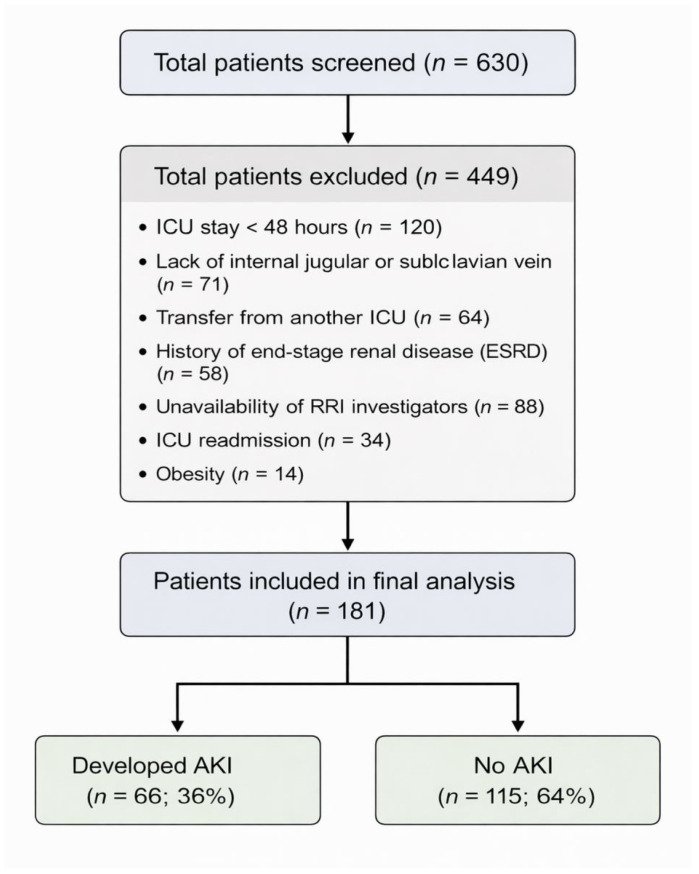
Study flow chart. Abbreviations: ICU, intensive care unit; RRI, renal resistive index; AKI, acute kidney injury; ESRD, end stage renal disease.

Figure 1 shows the study flowchart. Among 630 mechanically ventilated patients admitted to the ICU during the study period, 449 patients were excluded mainly because of an ICU stay of less than 48 h (*n* = 120), history of end-stage renal disease (*n* = 58), lack of an internal jugular or subclavian vein (*n* = 71), obesity (*n* = 14), transfer from another ICU (*n* = 64) or ICU readmission (*n* = 34), and unavailability of investigators who performed RRI (*n* = 88).

### 3.2. Baseline Characteristics

**Figure 2:**
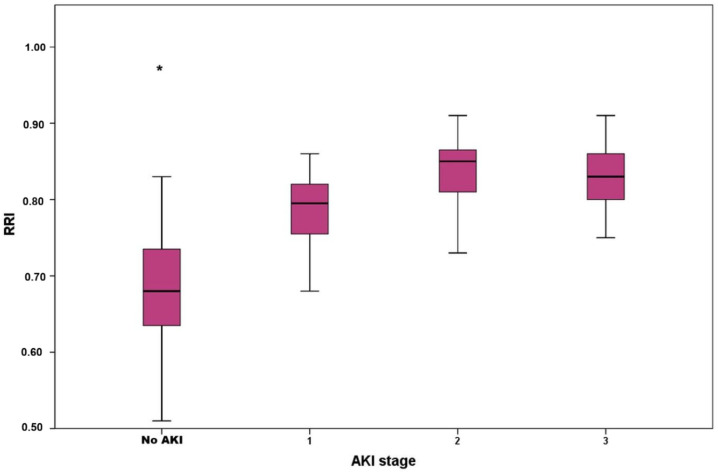
Boxplots of renal resistive index (RRI) according to acute kidney injury (AKI) stages. * *p* = 0.001 for no AKI vs. AKI stage 1, AKI stage 2 and AKI stage 3. No significant differences were found between AKI stages (Kruskal–Wallis test). Abbreviations: AKI, acute kidney injury; RRI, renal resistive index.

A total of 181 patients were included in the final analysis. Of them, 113 (62%) were males, and their median (IQR) age was 61 (44–73) years. The APACHE II score was 18 (15–22) and the SOFA score 8 (7–10). The most frequent comorbidities were hypertension (21%) and cardiovascular disease (20%). Sepsis was present in 43% and shock in 55% of patients on ICU admission. AKI occurred in 66 (36%) patients. Of them, 28 (15%) had AKI stage 1, 11 (6%) AKI stage 2 and 25 (14%) AKI stage 3. Figure 2 depicts the boxplots of RRI according to AKI stages; no significant differences were found between AKI stages. All-cause ICU mortality in the entire population was 27%, and CRRT was required by 12% of patients. Median RRI was 0.73 (0.65–0.80), Table 1. RRI, lactate, age, APACHE II and SOFA scores, the presence of sepsis and shock, as well as the history of hypertension and cardiovascular disease, were significantly higher in AKI compared with patients with no AKI, Table 1.

### 3.3. Correlations of RRI with Other Variables

**Figure 3:**
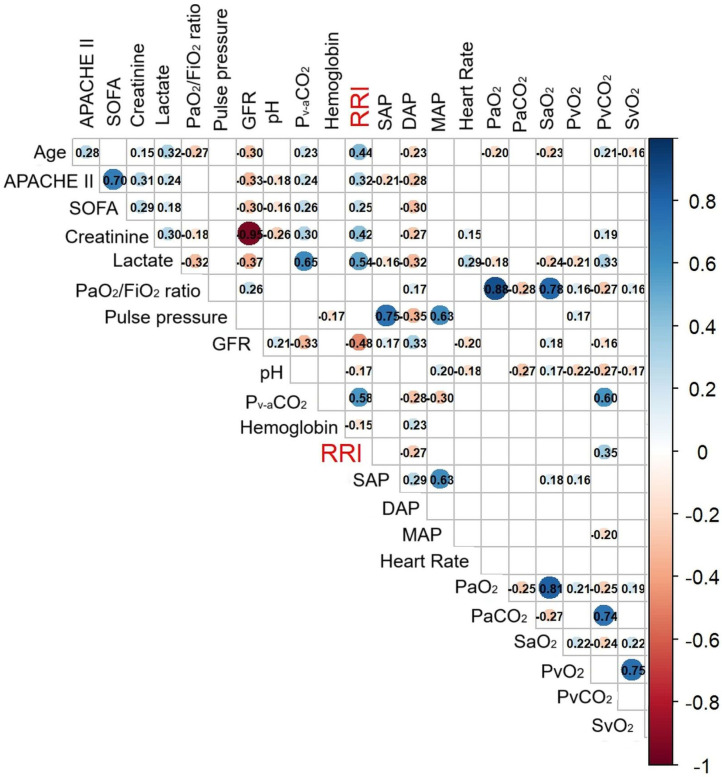
Correlation matrix of renal resistive index (RRI) with other variables. Numbers inside squares represent Spearman’s rho correlation coefficients. Blank squares denote non-significant correlations, e.g., *p* > 0.05. Abbreviations: APACHE, acute physiology and chronic health evaluation; SOFA, sequential organ failure assessment; SAP, systolic arterial pressure; DAP, diastolic arterial pressure; MAP, mean arterial pressure; GFR, glomerular filtration rate; RRI, renal resistive index; a, arterial; v, venous; v-a, veno-arterial difference; S, hemoglobin oxygen saturation.

Figure 3 demonstrates the correlation matrix of RRI with other variables. RRI was significantly correlated with age (r = 0.44, *p* = 0.001), APACHE II (r = 0.32, *p* = 0.001), SOFA (r = 0.25), lactate (r = 0.54, *p* = 0.001), GFR (r = −0.48, *p* = 0.001), and P_v-a_CO_2_ (r = 0.58, *p* = 0.001).

Multivariate models for AKI prediction

Table 2 shows the association of RRI with AKI development after adjustment with variables selected with LASSO regression. RRI was the only factor independently associated with AKI (OR 2.86 per 0.05 increase, 95% CI 1.64–4.98, *p* = 0.001). Table 3 shows the association of RRI with AKI stages after an ordinal multivariate logistic regression. RRI was strongly associated with higher odds of being in a higher AKI stage [OR per 0.05 increase = 2.54 (1.69–4.98) *p* = 0.001)].

### 3.4. RRI Performance for AKI Prediction

A ROC analysis of RRI revealed an AUC of 0.89 (0.84–0.94) for AKI prediction. Table 4 demonstrates the performance characteristics of RRI for AKI prediction. An RRI cut-off value of 0.77 had a sensitivity of 0.83 and a specificity of 0.82 for AKI prediction.

### 3.5. Mediation Analyses

**Figure 4:**
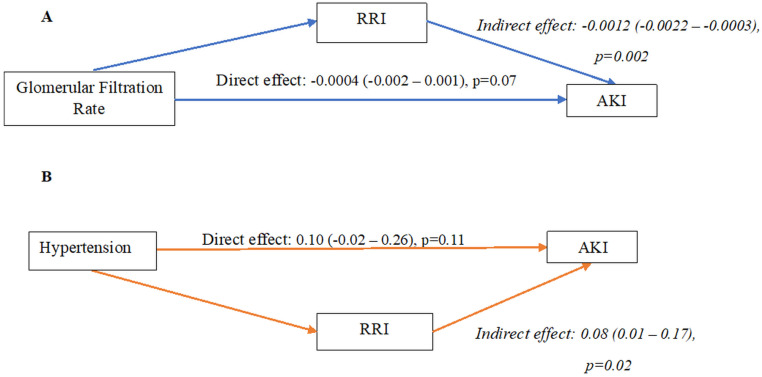
(**A**) Directed acyclic graph showing that renal resistive index (RRI) had a significant mediation effect on the association between baseline glomerular filtration rate (GFR) and acute kidney injury (AKI), highlighted by the significant indirect effect. (**B**) Directed acyclic graph showing that renal resistive index (RRI) had a significant mediation effect on the association between history of hypertension and acute kidney injury (AKI), highlighted by the significant indirect effect. *Abbreviations:* AKI, acute kidney injury; RRI, renal resistive index; GFR, glomerular filtration rate.

Since chronic kidney disease and hypertension are well known risk factors for AKI, as well as factors influencing RRI, we sought to find whether their effect on AKI development was mediated by RRI. We performed two mediation analyses using baseline GFR and history of hypertension as the exposures, respectively, and RRI as the mediator for AKI. Figure 4 presents the relevant directed acyclic graphs. Mediation analyses demonstrated that RRI significantly mediated the relationship between both GFR and hypertension with AKI development. Specifically, the indirect effect of GFR on AKI through RRI was significant (indirect effect = −0.0012, 95% CI −0.0022 to −0.0003, *p* = 0.002), while the direct effect was not (*p* = 0.07), indicating that the association between lower GFR and higher AKI risk was largely explained by RRI. Similarly, RRI significantly mediated the association between hypertension and AKI (indirect effect = 0.08, 95% CI 0.0–0.17, *p* = 0.02), whereas the direct effect of hypertension on AKI was not significant (*p* = 0.11). These findings suggest that RRI acts as an intermediate mechanism linking both reduced GFR and hypertension to AKI in critically ill patients.

### 3.6. Reclassification Tests

Table 5 shows the performance of RRI for AKI prediction. RRI, when added to the reference model built with LASSO regression, resulted in significantly higher ΔAUCs (DeLong *p* = 0.049), as well as in better reclassification of patients (total NRI = 0.52, *p* < 0.001).

### 3.7. Decision Curve Analysis

**Figure 5:**
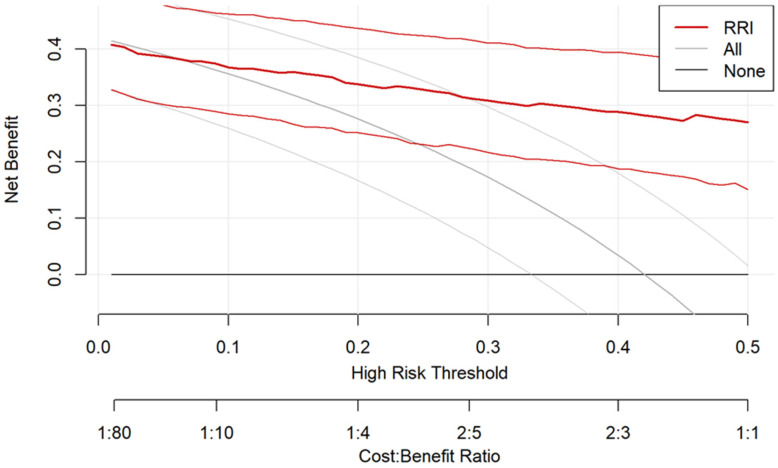
Decision curve analysis for the RRI model. The y-axis indicates the net benefit, calculated by summing the benefits (true positives) and subtracting the harms (false positives). The thick red line represents the RRI model, while the thin red lines represent the 95% confidence intervals. The solid grey line represents the strategy of treating all patients (assuming all are high risk), and the horizontal black line at the origin represents the strategy of treating no patients. The RRI model provides the highest net benefit across the clinical threshold range of 0.0 to 0.5, indicating superior clinical utility compared with default strategies. Abbreviations: RRI, renal resistive index.

Decision curve analysis demonstrated that the use of RRI to guide clinical decisions provided greater net benefit than the default strategies of treating all patients or treating none across a wide range of threshold probabilities (Figure 5). The RRI curve remained consistently above both reference strategies across most clinically relevant thresholds, indicating potential clinical utility in identifying patients at risk of AKI. The benefit was particularly evident across intermediate threshold probabilities, where the model yielded higher net benefit than alternative strategies.

### 3.8. Subgroup Analyses

**Figure 6:**
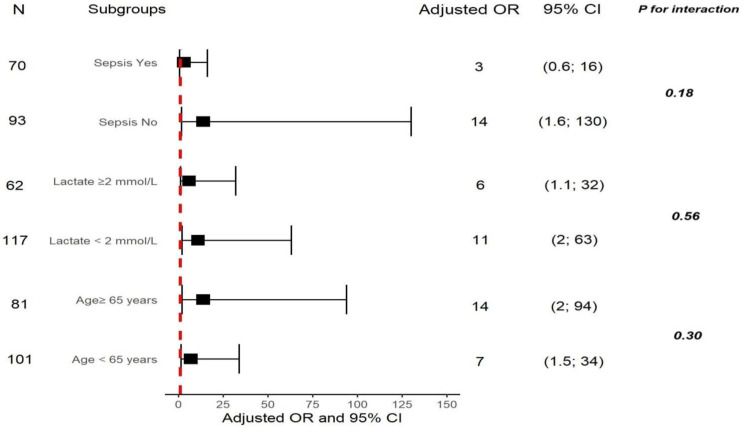
Forest plot of subgroup analyses for the association of renal resistive index (RRI) with acute kidney injury (AKI) development. Dotted red line represents odds ratio = 1. All models were adjusted for APACHE II, lactate, glomerular filtration rate, pH, pulse pressure, P_v-a_CO_2_, and hypertension. Interactions between RRI and stratification variables were examined using likelihood ratio tests. Abbreviations: RRI, renal resistive index; AKI, acute kidney injury; OR, odds ratio; CI, confidence interval; APACHE, acute physiology and chronic health evaluation; P_v-a_CO_2_, veno-arterial difference of carbon dioxide partial pressure.

We also performed a risk stratification analysis of RRI for the primary endpoint across multiple subgroups, e.g., according to age, lactate and sepsis. RRI was significantly associated with increased AKI development in subgroups defined by age < 65 years, age ≥ 65 years, lactate < 2 mmol/L, lactate ≥ 2 mmol/L, and presence or absence of sepsis. Significant interactions were not found between RRI and the above-mentioned variables (all *p* for interaction > 0.05). Figure 6 shows the forest plot of subgroup analyses for the association of RRI with AKI development.

## 4. Discussion

In this study, we evaluated the effectiveness of RRI on ICU admission for AKI prediction in a cohort of diverse ICU patient population. The main findings are the following: (i) RRI was an independent risk factor for both AKI development and AKI severity, (ii) RRI showed significant prognostic ability for AKI, and (iii) RRI was a mediator of both hypertension and low baseline GFR, regarding their effect on AKI development.

To reveal the association of RRI with AKI we undertook various approaches. More specifically, a multivariable logistic regression showed that RRI was the only independent predictor for AKI development, whereas when it was added to a reference model it resulted in better reclassification of patients, as revealed by NRI. Furthermore, a ROC analysis of RRI revealed high discriminative ability for AKI prediction. Decision curve analysis suggested that RRI may have meaningful clinical utility for identifying patients at increased risk of AKI. Across a broad range of clinically relevant threshold probabilities, the use of RRI resulted in a higher net benefit compared with strategies of treating all patients or none. These results are consistent with several prior investigations that have highlighted RRI as a valuable early marker of renal dysfunction in critically ill patients. For instance, several studies have demonstrated that elevated RRI values were independently associated with subsequent AKI in ICU cohorts [6,9,19]. Additionally, our findings are in line with the results of previous studies, which have reported excellent performance for RRI in predicting AKI in ICU patients with sepsis [5,20,21]. Similarly, a meta-analysis by Wei et al. [22] confirmed that RRI provides useful diagnostic and prognostic information regarding AKI. On the other hand, previous studies have shown the association of RRI with advanced AKI stages [19,23], consistent with the results of our multivariable ordinal regression analysis.

Our results emphasize that RRI’s predictive value remained consistent across diverse subgroups, including septic and non-septic patients, different levels of lactate, and different age categories. This consistency underlines its potential applicability as a bedside tool for early AKI risk stratification. Furthermore, the non-invasive and repeatable nature of Doppler-based RRI assessment makes it particularly appealing for dynamic monitoring of renal perfusion in the ICU setting. From a pathophysiological perspective, RRI is thought to reflect the resistance to arterial inflow, vascular compliance, downstream impedance, and intrarenal vascular tone or vasoconstriction. Increases in RRI may arise from elevated intrarenal vascular resistance (e.g., due to vasoconstriction, edema, or interstitial pressures), reduced arterial compliance, or alterations in downstream microcirculation [24]. Because these alterations may precede overt impairment of glomerular filtration, an elevated RRI could serve as an early warning metric for evolving AKI.

Although systemic hemodynamic variables such as pulse pressure or lactate provide information about global circulatory status, they may not accurately reflect renal microvascular perfusion. In contrast, RRI directly reflects intrarenal vascular resistance and may therefore capture early alterations in renal perfusion that are not detectable through systemic parameters alone [24]. In our study, RRI remained independently associated with AKI after adjustment for conventional hemodynamic and metabolic variables, suggesting that it may provide complementary information beyond routinely measured clinical parameters.

Nevertheless, not all studies have demonstrated equally strong predictive ability. Some investigations reported only moderate AUCs for RRI in AKI prediction, ranging from 0.70 to 0.76 [23,25,26], while others found limited or no predictive value in certain clinical settings [27,28,29]. These discrepancies likely reflect heterogeneity in patient populations, timing of RRI measurement, hemodynamic instability, pre-existing renal impairment, and differences in echography technique. Our study, which standardized timing (within 24 h of admission) and ensured hemodynamic stabilization before measurement, may partly explain the stronger predictive performance observed.

RRI should also be interpreted in the context of other established biomarkers of AKI. Several studies have compared RRI with biochemical markers such as neutrophil gelatinase-associated lipocalin (NGAL), cystatin C, interleukin-18 (IL-18), kidney injury molecule-1 (KIM-1), and the cell-cycle arrest biomarkers TIMP-2 and IGFBP7. In septic ICU patients, RRI demonstrated excellent performance for early AKI detection and even outperformed NGAL in predicting AKI development (AUC 0.90) in one prospective study [5]. Similarly, RRI showed comparable diagnostic accuracy to inflammatory and tubular injury biomarkers such as IL-18 and KIM-1 for predicting AKI within 24 h of admission (AUC 0.88) [20]. Other studies have reported similar predictive ability between RRI and urinary biomarkers such as NGAL or monocyte chemotactic protein 1 (MCP-1), although urinary biomarkers tended to demonstrate greater sensitivity for severe AKI, while RRI exhibited higher specificity [23]. Furthermore, studies evaluating cell-cycle arrest biomarkers have suggested that combining RRI with markers such as tissue inhibitor of metalloproteinase-2 × insulin-like growth factor-binding protein 7 (TIMP-2 and IGFBP7) may improve predictive accuracy compared with either approach alone, highlighting the potential value of multimodal risk assessment strategies [8]. Taken together, these findings suggest that RRI has a diagnostic performance comparable to several established AKI biomarkers while offering the advantages of being non-invasive, repeatable at the bedside, and capable of reflecting real-time renal hemodynamics.

We showed that RRI, when added to a reference model, not only resulted in a high value of AUC for AKI prediction (0.936), but also significantly increased the AUC and the reclassification of the model. This is in line with the results of other studies, which have reported similar findings [30]. Taking the above into consideration, we could assume that RRI, when integrated into other models, might enrich their predictive accuracy. However, our reference clinical model already demonstrated high discrimination (AUC 0.87 after bootstrapping), and the small ΔAUC of 0.046 (after bootstrapping) after the addition of RRI, although significant, could not actually represent clinically meaningful improvement (likely due to small sample size, selection bias, etc.).

We found that RRI had a significant mediation effect on the association between both low baseline GFR and hypertension, and AKI development. To the best of our knowledge, such a mediation effect is first described in the present study. These findings have a strong pathophysiological rationale, as chronic kidney disease (CKD) and hypertension are known risk factors for AKI [31,32,33]. Specifically, there is growing evidence suggesting that low GFR is associated with high RRI [34,35]. Likewise, hypertension has been found to be related to RRI [36]. From a pathophysiological perspective, RRI reflects not only intrarenal vascular resistance but also systemic hemodynamic and vascular factors, such as arterial stiffness and pulse pressure. Its association with hypertension and reduced GFR suggests that RRI integrates both renal and systemic determinants of perfusion. Our mediation analysis revealed that RRI mediated the associations of both hypertension and baseline GFR with AKI development. The absence of significant direct effects alongside significant indirect effects supports the concept that the influence of these variables on AKI risk is primarily transmitted through changes in renal vascular resistance, as captured by RRI. These findings imply that microvascular dysfunction and impaired renal perfusion, reflected by elevated RRI, may represent the common pathway through which chronic vascular and renal conditions predispose to AKI in the ICU setting. Interestingly, confirming previous findings [14], a correlation between the venous-arterial CO_2_ gap (P_v-a)_CO_2_) and RRI was observed, further indicating the relationship between RRI and tissue hypoperfusion.

The present study has certain limitations. First, this is a single center study, which could limit the generalizability of the results. Second, the study design introduces potential selection bias. A substantial proportion of screened patients were excluded (449 out of 630), including 88 patients due to operator unavailability. Excluding weekend admissions and patients without specific vascular access could meaningfully affect external validity. This limits generalizability to a broader ICU population. Third, it lacks information on the trajectory of RRI and its association with AKI. Fourth, the requirement for invasive mechanical ventilation limits generalizability to the broader ICU population. Fifth, mediation analysis of observational data does not establish causality, while decision curve analysis evaluates potential clinical utility rather than causal effects, and the observed benefit should be interpreted cautiously. Sixth, the lack of inter-observer variability is another limitation. Additionally, we have to acknowledge the risk of type I error due to multiple testing in subgroup analyses. Finally, sample size estimation was not made a priori. However, this is a prospective study with consecutively screened patients for eligibility, and its sample size is relatively large.

From a clinical perspective, RRI could serve as an early bedside marker of renal perfusion impairment in critically ill patients. Identification of patients with elevated RRI may prompt closer monitoring of renal function and hemodynamics and encourage optimization of potentially modifiable factors such as intravascular volume status, mean arterial pressure, and avoidance of nephrotoxic medications.

## 5. Conclusions

The findings of the present study reinforce the growing body of evidence supporting RRI as an independent early predictor of AKI and its severity in critically ill patients. It was also found to be a mediator of both hypertension and low baseline GFR, regarding their effect on AKI development in ICU patients.

Taken together, these findings suggest that RRI could be a reliable AKI predictive tool, while offering the advantages of being non-invasive, repeatable at the bedside, and capable of reflecting real-time renal hemodynamics.

In addition, patients with elevated RRI may benefit from more frequent renal function monitoring and early preventive strategies aimed at reducing the risk of acute kidney injury.

Future studies should further validate its utility, define optimal cut-off values across clinical contexts, and explore whether integrating RRI into multimodal predictive models can improve clinical decision making and outcomes.

## Figures and Tables

**Table 1:** Baseline characteristics for all patients and for those with and without AKI.

Characteristic	Overall N = 181	No AKI N = 115	AKI N = 66	*p*-Value
Sex, male	113 (62%)	72 (63%)	41 (62%)	0.9
Age, years	61 (44, 73)	54 (42, 70)	72 (57, 76)	0.001
**Comorbidities**				
Hypertension	38 (21%)	16 (14%)	22 (33%)	0.002
Cardiovascular disease	36 (20%)	17 (15%)	19 (29%)	0.023
**Admission characteristics**				
Shock	98 (55%)	47 (42%)	51 (77%)	0.001
Sepsis	70 (43%)	32 (33%)	38 (58%)	0.001
**Illness severity**				
APACHE II score	18 (15, 22)	16 (14, 20)	20 (17, 25)	0.001
SOFA score	8 (7, 10)	8 (7, 9)	9 (8, 10)	0.001
**Outcomes**				
ICU outcome, death	47 (27%)	16 (15%)	31 (51%)	0.001
ICU-LOS, days	16 (8, 27)	16 (7, 28)	18 (10, 27)	0.5
**Admission diagnosis**				0.01
Medical	73 (40%)	43 (37%)	29 (44%)	
Surgical	81 (44%)	47 (41%)	34 (51%)	
Trauma	28 (15%)	25 (22%)	3 (4.5%)	
**Laboratory tests**				
Creatinine, mg/dL	0.9 (0.7, 1.2)	0.8 (0.6, 1.1)	1.2 (0.9, 1.9)	0.001
Lactate, mmol/L	1.5 (1.0, 2.5)	1.1 (0.9, 1.7)	2.5 (1.6, 4.0)	0.001
Hemoglobin, g/dL	10.5 (9.1, 11.9)	10.5 (9.4, 11.9)	10.5 (8.7, 12.0)	0.5
GFR, ml/min/1.73 m^2^	78 (50, 114)	96 (63, 126)	50 (34, 73)	0.001
**Hemodynamic parameters**				
Pulse pressure, mmHg	55 (42, 74)	56 (40, 74)	52 (45, 74)	>0.9
RRI	0.73 (0.65, 0.80)	0.68 (0.63, 0.74)	0.81 (0.77, 0.84)	0.001
Systolic arterial pressure, mmHg	130 (116, 142)	130 (120, 144)	125 (112, 138)	0.042
Diastolic arterial pressure, mmHg	72 (61, 80)	74 (64, 82)	65 (59, 74)	0.001
Mean arterial pressure, mmHg	79 (65, 92)	81 (65, 93)	78 (65, 86)	0.3
Heart rate, beats/min	80 (70, 99)	78 (70, 93)	87 (70, 100)	0.084
**Gas exchange**				
pH	7.38 (7.34, 7.42)	7.39 (7.36, 7.43)	7.36 (7.31, 7.41)	0.001
PaO_2_/FiO_2_ ratio	227 (149, 318)	246 (165, 354)	205 (145, 258)	0.009
PaCO_2_, mmHg	39 (36, 43)	39 (36, 42)	38 (36, 43)	0.9
SaO_2,_ %	98 (97, 98)	98 (97, 99)	98 (96, 98)	0.032
P_v_CO_2_, mmHg	45 (40, 50)	43 (39, 48)	47 (43, 52)	0.001
S_v_O_2_, %	78 (73, 83)	79 (73, 83)	78 (72, 82)	0.5
P_v-a_CO_2_, mmHg	3.7 (1.8, 10.2)	2.3 (1.5, 4.3)	10.2 (6.0, 14.1)	0.001

Data are expressed as median (interquartile range), unless otherwise indicated. Abbreviations: AKI, acute kidney injury; APACHE, acute physiology and chronic health evaluation; SOFA, sequential organ failure assessment; ICU, intensive care unit; LOS, length of stay; GFR, glomerular filtration rate; RRI, renal resistive index; a, arterial; v, venous; v-a, veno-arterial difference; S, hemoglobin oxygen saturation.

**Table 2:** Multivariate logistic regression for AKI development.

Independent Variable	Odds Ratio	95% CI	*p* Value
Lactate	1.10	0.65–1.86	0.70
GFR	0.99	0.98–1.01	0.66
pH	0.00	0.01–13.44	0.14
Pulse pressure	0.97	0.93–1.00	0.06
P_v-a_CO_2_	1.11	0.96–1.28	0.14
APACHE II score	1.08	0.96–1.20	0.17
Hypertension	2.58	0.69–9.55	0.15
RRI, per 0.05 increase	2.86	1.64–4.98	0.001

Abbreviations: AKI, acute kidney injury; CI, confidence interval; RRI, renal resistive index; P_v-a_CO_2_, veno-arterial carbon dioxide partial pressure; APACHE, acute physiology and chronic health evaluation; GFR, glomerular filtration rate.

**Table 3:** Multivariate ordinal logistic regression with AKI stages as the dependent variable.

Variable	Odds Ratio	95% CI	*p*-Value
pH	0.002	0.001–0.006	0.001
RRI, per 0.05 increase	2.54	1.69–3.83	0.001
P_v-a_CO_2_	1.21	1.09–1.34	0.001
Pulse pressure	0.98	0.95–0.99	0.04
APACHE II	1.07	0.99–1.17	0.09
Lactate	0.87	0.71–1.07	0.19
GFR	0.99	0.98–1.01	0.24
Hypertension	1.43	0.54–3.76	0.47

Abbreviations: AKI, acute kidney injury; CI, confidence interval; RRI, renal resistive index; P_v-a_CO_2_, veno-arterial carbon dioxide partial pressure; APACHE, acute physiology and chronic health evaluation; GFR, glomerular filtration rate.

**Table 4:** Performance characteristics of RRI for AKI prediction.

Metric	Value (95% CI)
ROC-AUC	0.890 (0.840–0.941)
* Cut-off RRI value	0.77
Sensitivity	0.83 (0.75, 0.90)
Specificity	0.82 (0.70, 0.90)
Positive predictive value	0.89 (0.81, 0.94)
Negative predictive value	0.74 (0.62, 0.84)
Positive likelihood ratio	4.59 (2.73, 7.71)
Negative likelihood ratio	0.20 (0.13, 0.31)

* Determined according to Youden’s index. Abbreviations: AKI, acute kidney injury; CI, confidence interval; RRI, renal resistive index; ROC, receiver operating characteristic; AUC, area under curve.

**Table 5:** Performance of RRI for AKI prediction.

Reference Model Selected by LASSO Regression	Added Baseline Variable	AUC of the New Model	ΔAUC	DeLong Test *p* Value	Total NRI (95% CI)	*p* Value
Hypertension + APACHE II + Lactate + Pulse Pressure + GFR + pH + P_v-a_CO_2_ AUC (95% CI): 0.897 (0.843–0.951)						
	RRI	0.936	0.039	0.049	0.52 (0.27–0.77)	0.001
					
					

Abbreviations: AKI, acute kidney injury; RRI, renal resistive index; LASSO, least absolute shrinkage and selection operator; AUC, area under curve; CI, confidence interval; Δ, difference; NRI, net reclassification improvement; APACHE, acute physiology and chronic health evaluation; GFR, glomerular filtration rate; P_v-a_CO_2_, veno-arterial difference of carbon dioxide partial pressure.

## Data Availability

The datasets used/or analyzed in the present study are available from the corresponding author on reasonable request.
